# Radiological Confirmation of Enteral Feeding Tube Placement Before Initiating Enteral Nutrition: A Review

**DOI:** 10.7759/cureus.115588

**Published:** 2026-09-01

**Authors:** Lavanya Chhetri, Pokhraj P Suthar, Keyur Parekh

**Affiliations:** 1 Department of Clinical Nutrition, Rush University, Chicago, USA; 2 Department of Diagnostic Radiology and Nuclear Medicine, Rush University Medical Center, Chicago, USA

**Keywords:** enteral nutrition, feeding tube, fluoroscopy, nasogastric tube, radiography, tube placement

## Abstract

Radiological confirmation of enteral feeding tube placement is widely regarded as the gold standard for verifying the position of blind-inserted feeding tubes before initiating enteral nutrition. Misplacement of feeding tubes into the respiratory tract or incorrect positioning within the gastrointestinal tract can lead to severe complications, including aspiration pneumonia, pneumothorax, pulmonary injury, and death. Although bedside methods such as pH testing are useful for ongoing monitoring, radiographic imaging remains the most accurate technique for confirming initial tube placement. This review summarizes the indications for radiological confirmation, imaging techniques, interpretation criteria, advantages, limitations, complications associated with incorrect placement, and current evidence-based recommendations.

## Introduction and background

Enteral nutrition (EN) is the preferred method of nutritional support for patients with a functional gastrointestinal tract who cannot meet their nutritional requirements orally [1]. Compared with parenteral nutrition, EN helps maintain gastrointestinal function, reduces infectious complications, and is associated with improved clinical outcomes [2]. It is commonly used in critically ill patients, individuals with neurological disorders, and those with conditions that impair swallowing [3].

The safe delivery of EN depends on accurate placement of feeding tubes such as nasogastric (NG), nasoduodenal (ND), and nasojejunal (NJ) tubes. Blind bedside insertion is widely practiced because it is simple and cost-effective; however, it carries a risk of tube malposition into the respiratory tract or incorrect placement within the gastrointestinal tract [4]. Such misplacement may result in serious complications, including aspiration pneumonia, pneumothorax, pulmonary injury, and even death [4].

Although bedside methods such as pH testing, aspiration of gastric contents, and assessment of external tube length can assist in tube placement assessment, they cannot reliably confirm correct initial positioning [5]. Current clinical guidelines recommend using validated methods for confirming initial tube placement before enteral feeding or medication administration. Gastric aspirate pH testing may be used as an initial confirmation method for NG tubes in appropriate patients, whereas radiographic confirmation is recommended when bedside confirmation is unsuccessful, unavailable, inconclusive, or when there is clinical concern regarding tube position [6]. Consequently, radiological confirmation, usually by plain chest and abdominal radiography, remains an established reference and definitive method for confirming tube position, particularly for excluding inadvertent respiratory placement [6]. Therefore, rather than describing radiography universally as the “gold standard,” it is more appropriate to recognize radiography as the definitive confirmation method when radiological verification is indicated. Proper interpretation of radiographs is essential to ensure the tube follows the correct anatomical pathway and terminates in the intended location [6]. Radiographic interpretation should assess the entire course of the tube, including whether it follows the expected anatomical pathway, remains outside the respiratory tract, and terminates at an appropriate location for the intended route of feeding [6,7]. Particular attention should be given to the tube trajectory and distal tip position before EN or medications are administered.

This review summarizes the role of radiological confirmation in enteral feeding tube placement, including its clinical indications, imaging techniques, interpretation criteria, advantages, limitations, potential complications of tube misplacement, and current evidence-based recommendations for improving patient safety.

## Review

Why radiological confirmation is necessary

Radiographic confirmation is recommended because it provides the most reliable method of verifying correct feeding tube placement before enteral feeding is initiated. It confirms that the tube has followed the esophagus rather than the tracheobronchial tree and verifies that the tip is positioned in the intended anatomical location, such as the stomach, duodenum, or jejunum. Radiographic imaging also enables the detection of tube coiling, kinking, or migration, as well as inadvertent pulmonary placement that may not be identified by bedside assessment methods alone. By accurately confirming tube position prior to use, radiological confirmation substantially reduces the risk of serious, and potentially fatal, complications associated with feeding through a misplaced tube [7].

Radiological techniques

Radiological techniques used for enteral feeding tube confirmation include plain chest and abdominal radiography (X-ray), fluoroscopy-guided tube placement, contrast radiography, computed tomography (CT), and point-of-care ultrasound (POCUS).

Plain Chest and Abdominal Radiography (X-Ray)

Plain chest and abdominal radiography remains the most widely accepted and frequently used imaging modality for confirming the position of blindly inserted enteral feeding tubes before the initiation of enteral nutrition. It is considered the gold standard because it provides direct visualization of the entire course of the feeding tube, allowing clinicians to accurately identify its anatomical location and detect inadvertent malposition. Unlike bedside verification methods, such as auscultation or pH testing, radiography offers a high degree of accuracy in distinguishing correct gastrointestinal placement from potentially hazardous respiratory placement. Consequently, most international clinical guidelines recommend radiographic confirmation following initial blind insertion of nasogastric, nasoduodenal, and nasojejunal feeding tubes, particularly in critically ill patients and those at increased risk of tube misplacement [8].

An adequately performed radiograph should clearly demonstrate the complete trajectory of the feeding tube from the nasal cavity, through the esophagus, across the diaphragm, and into the stomach or small intestine. Visualization of the entire tube is essential because incomplete imaging may obscure the actual location of the tube tip or fail to identify abnormal tube pathways. Correct interpretation requires careful assessment by trained healthcare professionals, as errors in radiograph interpretation have been associated with serious adverse events, including the administration of enteral nutrition into the respiratory tract. Therefore, both obtaining an appropriate radiograph and ensuring accurate interpretation are critical components of safe enteral feeding practice [9].

Several radiographic criteria have been established to confirm correct feeding tube placement. The tube should descend centrally through the esophagus without deviating into the trachea or main bronchi, indicating that it has followed the normal gastrointestinal pathway (Figure 1). It should cross the diaphragm in the midline before entering the stomach and should not remain above the diaphragm or follow the course of the respiratory tree. The distal tip of the tube must be located below the diaphragm, with its final position depending on the intended feeding route. For gastric feeding, the tube tip should terminate within the stomach, whereas for post-pyloric feeding, it should extend beyond the pylorus into the duodenum or proximal jejunum. Adherence to these radiographic criteria minimizes the risk of feeding-related complications and ensures the safe initiation of enteral nutrition [8,9].

**Figure 1:**
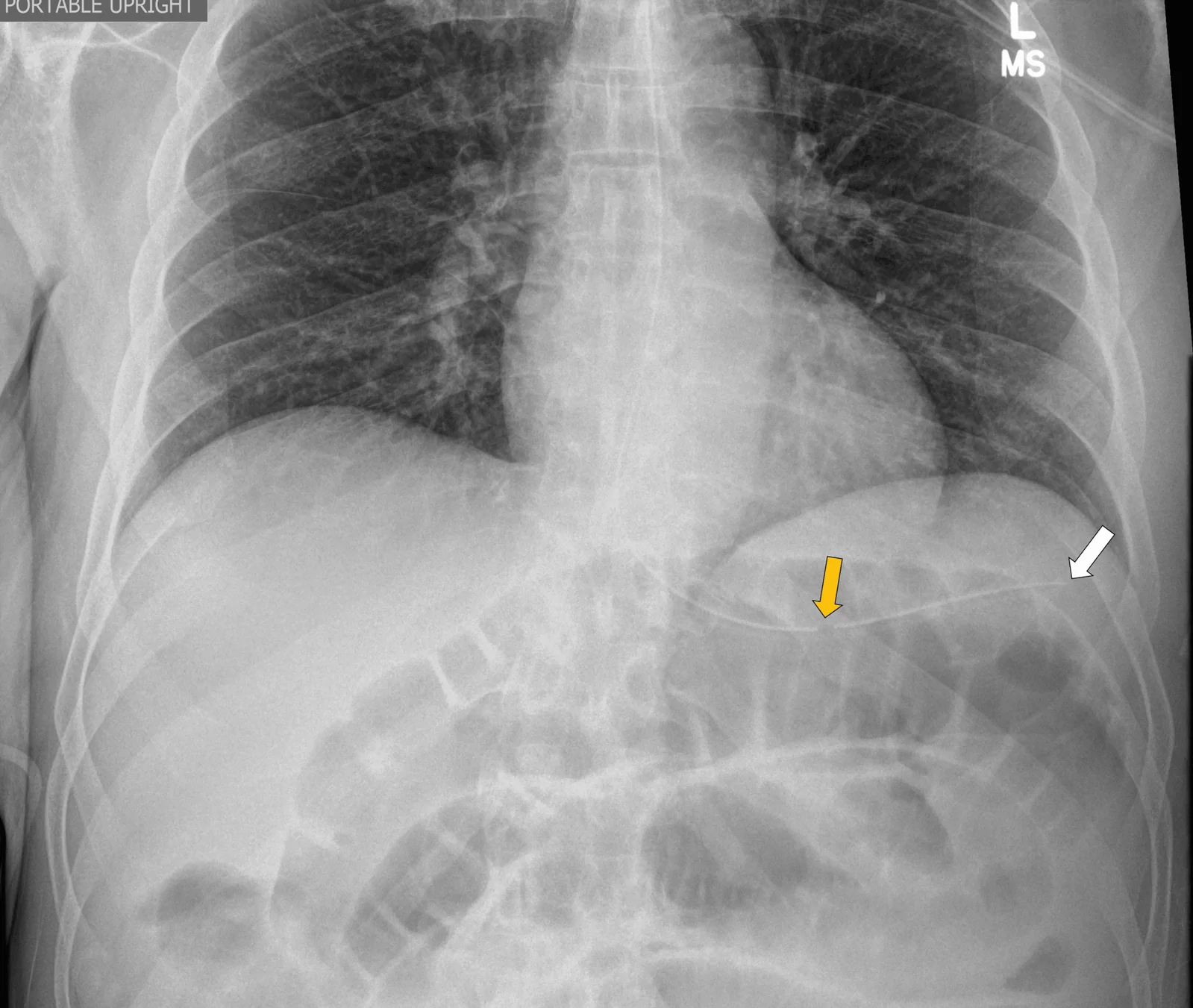
Portable frontal chest and abdominal radiograph demonstrating enteric tube positioning. The enteric tube is appropriately positioned, with the tip located within the stomach (white arrow) and the side port positioned below the level of the gastroesophageal junction (orange arrow). Multiple mildly dilated bowel loops are noted within the abdomen, consistent with the patient’s history of bowel obstruction.

Fluoroscopy-Guided Tube Placement

Fluoroscopy-guided tube placement is an image-guided technique that provides continuous real-time visualization during the insertion of enteral feeding tubes. Unlike blind bedside placement, fluoroscopy enables the operator to monitor the progression of the tube throughout the procedure, thereby improving the accuracy of placement and minimizing the risk of malposition. This technique is particularly valuable in patients in whom bedside insertion has been unsuccessful or when accurate post-pyloric tube placement is clinically indicated. It is also recommended for patients with altered upper gastrointestinal anatomy due to previous surgery, strictures, or anatomical abnormalities, as well as for individuals at high risk of repeated tube misplacement. By allowing direct visualization of the tube as it advances through the gastrointestinal tract, fluoroscopy facilitates successful placement while reducing procedural difficulties and complications [10-12].

The primary advantage of fluoroscopy-guided placement is its ability to provide real-time imaging, enabling immediate correction of the tube's direction if resistance or malposition occurs during insertion. This results in a high success rate for accurate tube placement, particularly for nasoduodenal and nasojejunal feeding tubes that require advancement beyond the pylorus. Furthermore, the technique decreases the likelihood of repeated insertion attempts, thereby improving patient comfort, shortening procedure time, and reducing delays in the initiation of enteral nutrition. These benefits make fluoroscopy a valuable option in patients with complex clinical conditions or when blind placement techniques are likely to fail [10-12].

Despite its advantages, fluoroscopy-guided tube placement has several limitations. The procedure exposes both patients and healthcare personnel to ionizing radiation, although the radiation dose is generally low when appropriate protocols are followed. In addition, fluoroscopy is associated with higher costs than conventional bedside placement because it requires specialized imaging equipment, dedicated radiology facilities, and trained personnel to perform and interpret the procedure. Limited availability in some healthcare settings and the need to transport critically ill patients to the radiology department may also restrict its routine use. Consequently, fluoroscopy is generally reserved for selected patients in whom blind placement is unsuccessful or when precise post-pyloric tube placement is essential [10-12].

Contrast Radiography

Contrast radiography is an adjunctive imaging technique used when the position of an enteral feeding tube remains uncertain after standard radiographic assessment or when there is concern for gastrointestinal perforation [13]. In this procedure, a small amount of water-soluble contrast agent is administered through the feeding tube before radiographic imaging. The contrast medium outlines the gastrointestinal lumen, allowing clear visualization of the tube's course and precise localization of its distal tip (Figure 2) [14]. This technique is particularly useful in situations where the tube tip is difficult to identify on plain radiographs due to overlapping anatomical structures, poor image quality, or complex patient anatomy. It can also assist in differentiating intragastric, post-pyloric, or inadvertently misplaced tubes, thereby facilitating appropriate clinical decision-making [14].

**Figure 2:**
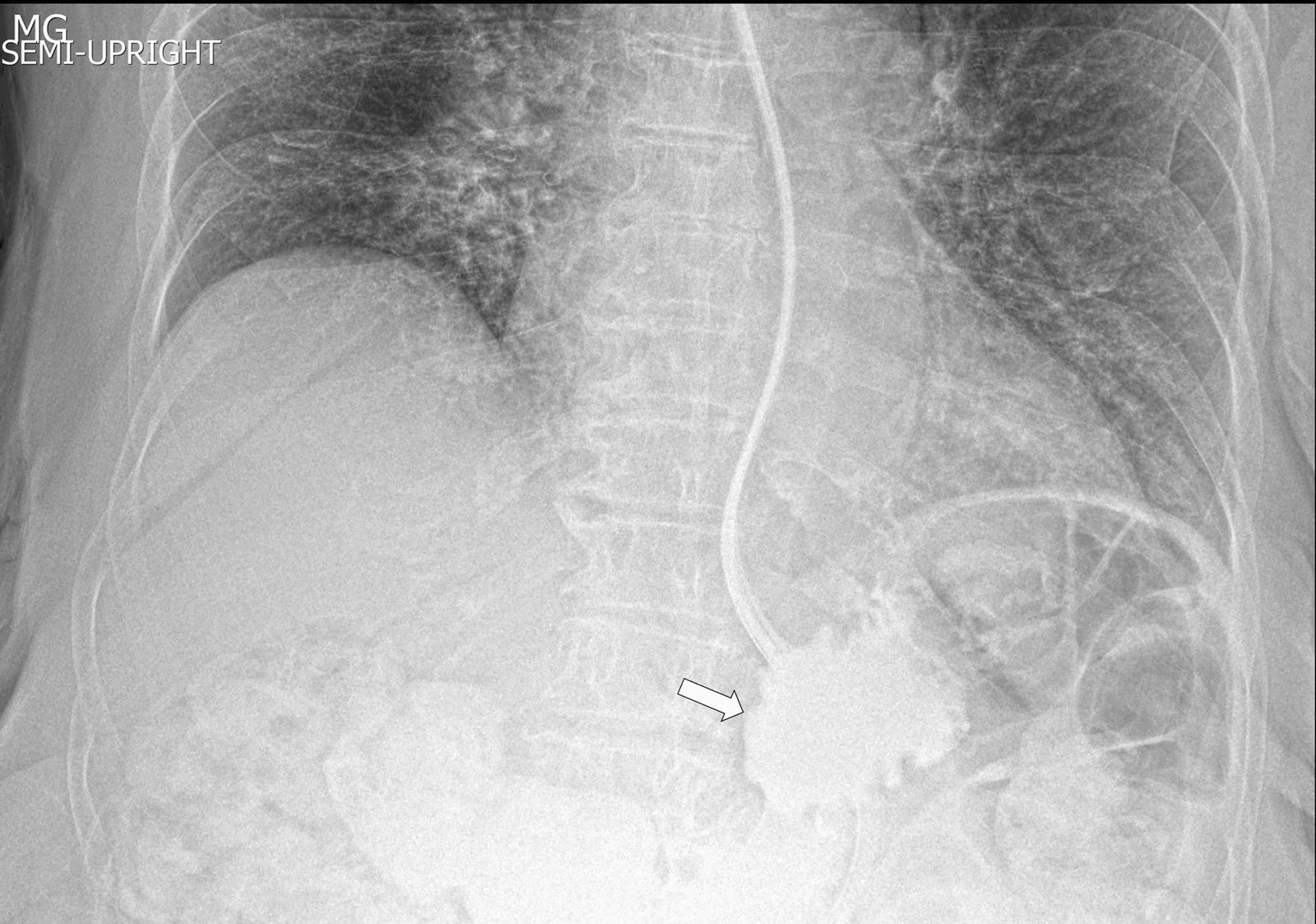
Portable chest and abdominal contrast radiograph demonstrating appropriate positioning of an enteric tube. The enteric tube is appropriately positioned within the stomach, with administered radiopaque contrast visualized filling the gastric lumen (white arrow).

An important advantage of contrast radiography is its ability to improve diagnostic accuracy when conventional radiographs are inconclusive. The use of water-soluble contrast enhances visualization of the gastrointestinal tract and helps confirm whether the feeding tube has reached the intended anatomical location. In addition, when gastrointestinal perforation is suspected, water-soluble contrast agents are preferred over barium because they are less likely to cause severe inflammatory complications if leakage into the peritoneal cavity occurs [15]. Consequently, contrast radiography serves as a valuable supplementary imaging modality in selected clinical situations where standard radiographic confirmation is insufficient.

Despite its diagnostic benefits, contrast radiography is not routinely recommended for all patients undergoing enteral feeding tube placement. The procedure requires additional imaging, administration of contrast material, and may delay the initiation of enteral nutrition. Furthermore, although water-soluble contrast agents are generally considered safe, they should be used cautiously in patients at high risk of aspiration, as aspiration of hyperosmolar contrast material may lead to pulmonary complications. Therefore, contrast radiography is typically reserved for cases in which tube position cannot be confidently established by plain radiography or when gastrointestinal perforation or other complications are suspected [16].

Computed Tomography (CT) and Point-of-Care Ultrasound (POCUS)

CT and POCUS are not routinely used for initial enteral feeding tube confirmation. CT is generally reserved for complex cases or suspected complications due to its higher radiation exposure, cost, and limited suitability for routine verification, while POCUS is an emerging bedside technique that is highly operator-dependent and currently lacks sufficient accuracy for universal replacement of radiographic confirmation.

The radiological techniques used for enteral feeding tube confirmation are presented in Table 1.

**Table 1:** Radiological techniques used for enteral feeding tube confirmation.

Radiological technique	Principle	Main indications	Advantages	Limitations	Radiation exposure	Role in clinical practice
Plain radiography (chest/abdominal X-ray)	Two-dimensional X-ray visualizes the entire course of the feeding tube from the nose to its tip.	Initial confirmation of blind-inserted nasogastric (NG), nasoduodenal (ND), and nasojejunal (NJ) tubes before feeding.	Gold standard for initial verification; widely available; detects tube malposition, coiling, pneumothorax, and pulmonary placement.	Requires trained interpretation; may delay feeding; exposes patients to ionizing radiation; repeat imaging may be required if tube migrates.	Low	First-line imaging modality recommended by major guidelines for confirming initial tube placement.
Fluoroscopy-guided placement	Continuous real-time X-ray imaging guides tube advancement into the desired gastrointestinal location.	Difficult tube insertions, post-pyloric feeding, altered gastrointestinal anatomy, repeated failed bedside placement.	High placement success; real-time visualization; minimizes repeated insertion attempts; accurate post-pyloric placement.	Requires radiology suite, specialist staff, higher cost, and longer procedure time.	Moderate	Preferred when bedside placement is unsuccessful or precise post-pyloric positioning is required.
Contrast-enhanced radiography	Administration of water-soluble contrast medium outlines the gastrointestinal tract and tube tip.	Uncertain tube position; suspected gastrointestinal perforation; confirmation of gastrostomy or jejunostomy tubes in selected cases.	Clearly demonstrates tube tip location and contrast flow; detects leakage or perforation.	Additional procedure; contrast allergy (rare); not routinely required for uncomplicated tube placement.	Low–moderate	Reserved for selected diagnostic situations rather than routine confirmation.
Computed tomography (CT)	Cross-sectional imaging provides high-resolution visualization of the tube and surrounding anatomy.	Complex anatomy, suspected complications (e.g., perforation, abscess), inconclusive radiographs.	Highest anatomical detail; identifies associated thoracic or abdominal complications.	High radiation dose; expensive; not suitable for routine tube confirmation; limited availability in emergencies.	High	Used only when complications or uncertain anatomy require further evaluation.
Ultrasound (point-of-care ultrasound, POCUS)	Real-time ultrasonography visualizes the tube in the esophagus or stomach without ionizing radiation.	Bedside assessment in critically ill patients, children, pregnancy, or when repeated radiation should be avoided.	No radiation; portable; immediate results; can reduce need for confirmatory X-rays in experienced hands.	Operator-dependent; reduced image quality in obesity or bowel gas; lower accuracy for post-pyloric placement.	None	Emerging adjunct to radiography but not yet a universal replacement for initial confirmation.

Radiological signs of malposition

Radiographic evaluation plays a crucial role not only in confirming correct enteral feeding tube placement but also in identifying tube malposition before the initiation of enteral nutrition [7]. Careful assessment of the entire course of the tube on chest and abdominal radiographs is essential, as incorrect placement may not be clinically apparent and can lead to serious or fatal complications if feeding is commenced. Recognition of abnormal tube pathways and tip locations enables prompt removal or repositioning of the tube, thereby preventing patient harm.

One of the most serious forms of malposition is inadvertent entry of the feeding tube into the tracheobronchial tree [17]. On radiographs, the tube may deviate from the midline and enter either the right or left main bronchus instead of descending through the esophagus. The right main bronchus is more commonly affected because of its wider diameter and more vertical orientation (Figure 3) [18]. If the tube advances further into the bronchial tree, it may terminate within the lung parenchyma (pulmonary placement), significantly increasing the risk of pulmonary complications such as aspiration, pneumothorax, pulmonary hemorrhage, lung perforation, or the accidental administration of enteral feeds into the respiratory tract [19].

**Figure 3:**
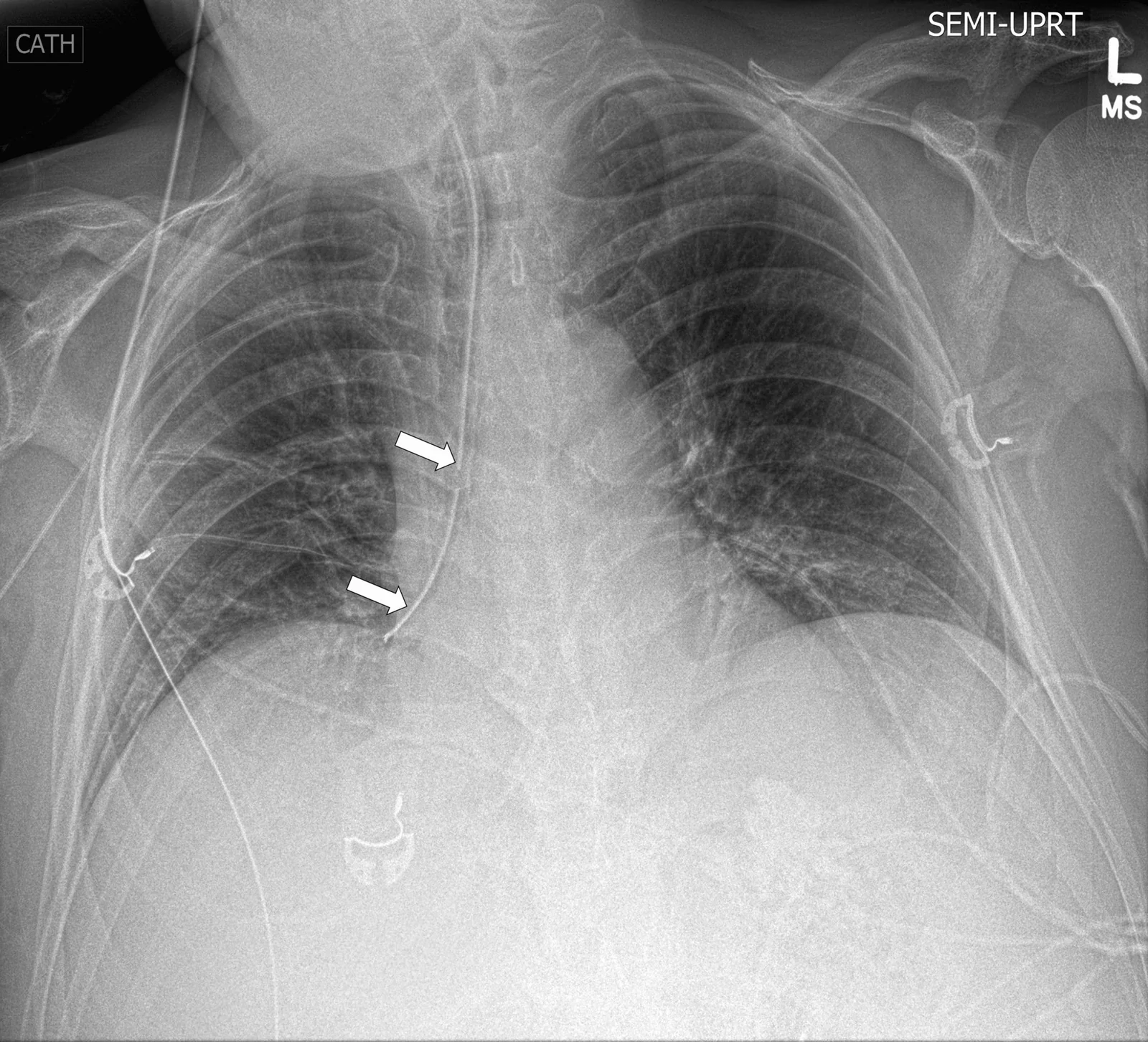
Portable frontal chest and abdominal radiograph demonstrating malposition of an enteric tube. The enteric tube has inadvertently entered the right main bronchus (white arrow) instead of passing through the esophagus into the stomach.

Radiographs may also demonstrate pleural placement, in which the feeding tube perforates the lung or airway and enters the pleural cavity. This rare but potentially life-threatening complication is often associated with pneumothorax, hydropneumothorax, pleural effusion, or empyema. Immediate recognition and appropriate management are essential to prevent further morbidity and mortality.

Another common radiographic finding is coiling of the feeding tube within the esophagus (Figure 4)[20]. This usually occurs when the tube encounters resistance during insertion and fails to progress into the stomach. Similarly, the tube may loop within the pharynx or oropharynx, particularly in patients with impaired swallowing, altered consciousness, or difficult upper airway anatomy. These malpositions prevent effective enteral feeding and increase the risk of aspiration if they are not identified before use.

**Figure 4:**
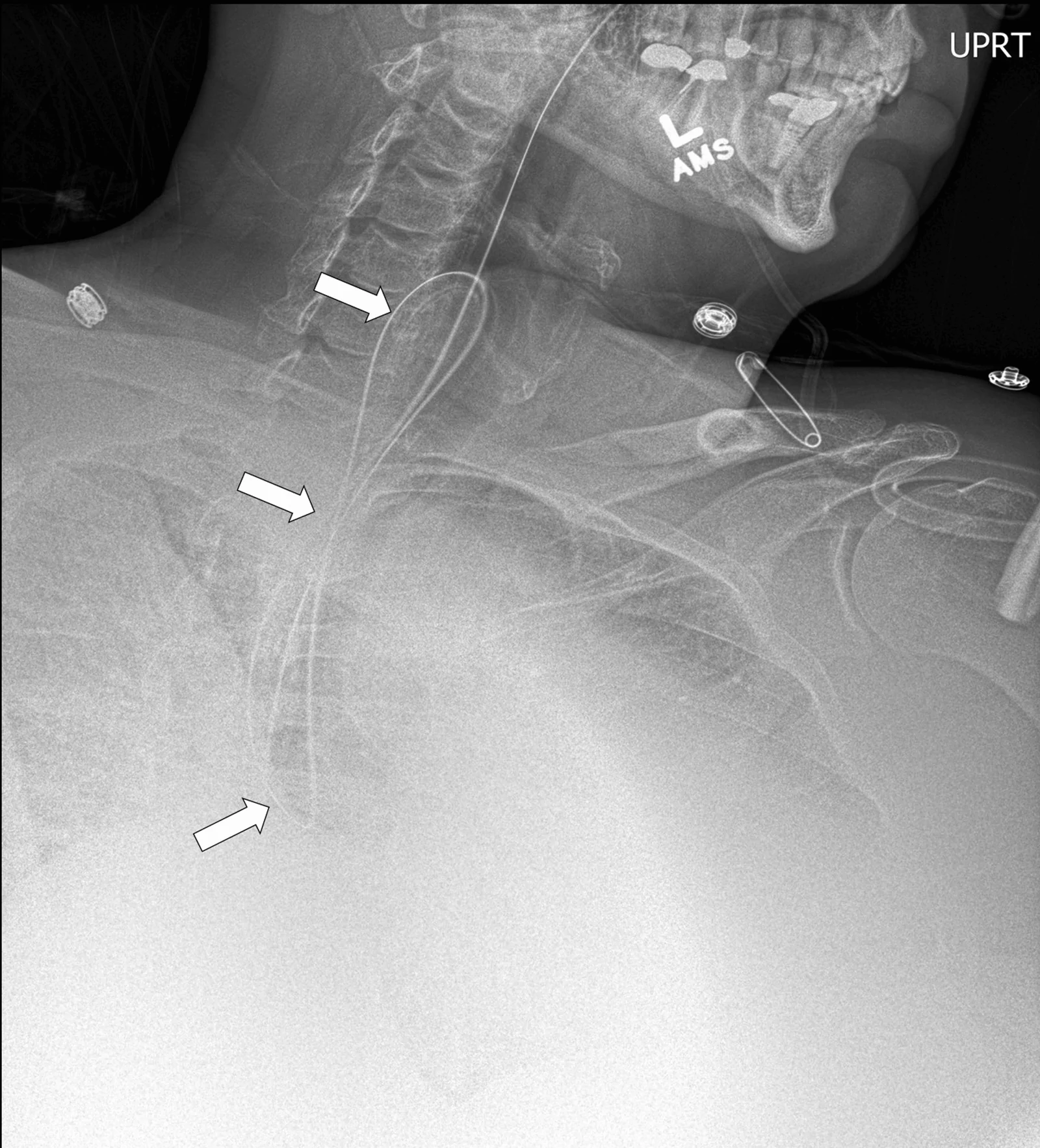
Portable chest and abdominal radiograph demonstrating malposition of an enteric tube. The enteric tube is coiled within the upper to mid esophagus (white arrow) and has failed to advance through the esophagus into the stomach.

Incorrect tube tip position may also be identified when the distal end remains above the gastroesophageal junction instead of extending below the diaphragm into the stomach or small intestine (Figure 5)[21]. A tube terminating in the distal esophagus is associated with an increased risk of gastroesophageal reflux and aspiration of gastric contents, making it unsuitable for enteral nutrition [22]. Therefore, the tube tip should always be confirmed below the diaphragm and within the intended feeding location before feeding is initiated.

**Figure 5:**
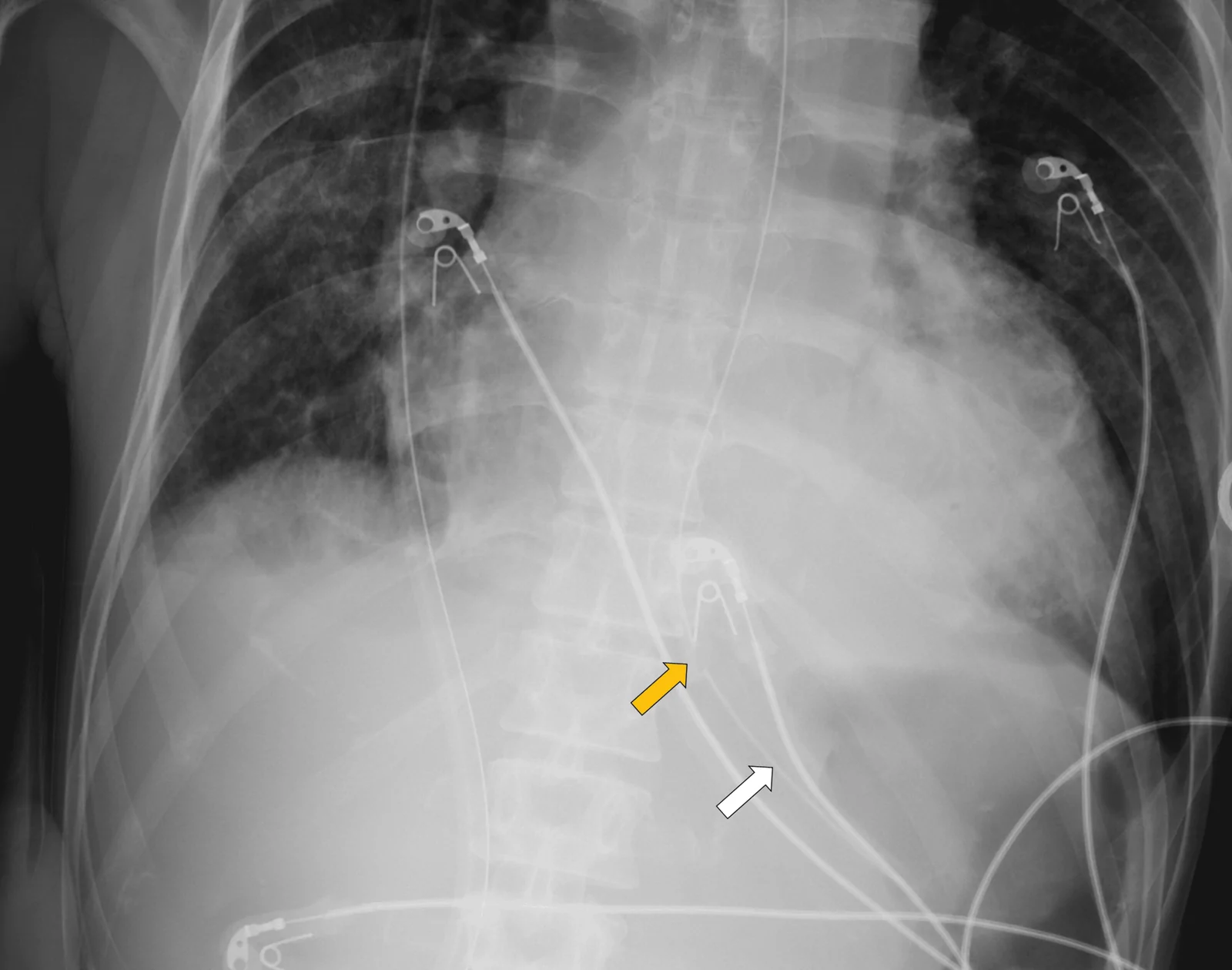
Portable chest and abdominal radiograph demonstrating suboptimal positioning of an enteric tube. The enteric tube tip is located within the stomach (white arrow); however, the side port remains at the level of the gastroesophageal junction (orange arrow). Feeding should be withheld until the tube is advanced by at least 5 cm to ensure the side port is positioned within the stomach and reduce the risk of aspiration.

A very rare but severe complication is intracranial placement of the feeding tube, which is primarily reported in patients with skull base or severe facial trauma [23,24]. In these cases, the tube may inadvertently pass through a fracture in the cribriform plate or skull base and enter the cranial cavity. As this complication carries an extremely high risk of neurological injury and death, blind nasal tube insertion should be avoided in patients with suspected basal skull fractures until appropriate imaging has excluded this possibility.

Early recognition of these radiological signs of malposition is essential because initiating enteral nutrition through an incorrectly positioned feeding tube can result in catastrophic complications, including aspiration pneumonia, pneumothorax, pleural infusion of enteral formula, severe pulmonary injury, sepsis, and death. Careful interpretation of radiographs by appropriately trained healthcare professionals is therefore a fundamental component of patient safety and quality care in enteral nutrition practice.

Complications associated with incorrect placement

Failure to identify feeding tube malposition before the initiation of enteral nutrition can result in serious, and sometimes fatal, complications. Incorrect placement may lead to pulmonary, gastrointestinal, and nutritional consequences that significantly increase patient morbidity, prolong hospitalization, and raise healthcare costs. Prompt radiological confirmation before feeding is therefore essential to minimize these risks and ensure safe enteral nutrition.

Pulmonary Complications

Pulmonary complications are among the most common and severe consequences of feeding tube malposition, particularly when the tube is inadvertently inserted into the tracheobronchial tree [25]. In a review of 9,931 narrow-bore nasoenteric tube placements, 1.9% were malpositioned in the tracheobronchial tree; among these malpositions, pneumothorax occurred in approximately 18.7%, and at least five deaths were attributed to the complication [26]. Another clinical series reported respiratory-tree misplacement in 3.2% of patients, with pneumothorax occurring in 1.2% and death in 0.5% [27]. Administration of enteral nutrition through a tube positioned within the respiratory tract can result in aspiration pneumonia, which is associated with significant morbidity and mortality, especially in critically ill and mechanically ventilated patients [28]. In some cases, advancement of the tube into the lung parenchyma may cause pulmonary trauma, leading to pulmonary hemorrhage or lung perforation. Accidental perforation of the pleura may also result in pneumothorax or hydrothorax, requiring urgent intervention such as chest tube insertion. Repeated trauma or severe injury to the airway may lead to the development of a bronchopleural fistula, a rare but serious complication associated with persistent air leakage and respiratory compromise. Collectively, these complications can progress to acute respiratory failure, necessitating intensive care support and significantly worsening patient outcomes.

Gastrointestinal Complications

Incorrect tube placement may also cause significant gastrointestinal injury. Forceful advancement of the feeding tube can result in esophageal perforation, particularly in patients with esophageal strictures, diverticula, or previous upper gastrointestinal surgery [28]. Although less common, gastric perforation may occur if excessive pressure is applied during insertion or if the stomach wall is weakened by underlying pathology. Advancement beyond the intended location may also cause injury to the duodenum or small bowel, occasionally resulting in bowel perforation. Leakage of gastrointestinal contents into the peritoneal cavity following perforation can lead to peritonitis, a life-threatening condition requiring prompt diagnosis and surgical management. Early recognition of these complications is essential to prevent severe infection, sepsis, and further clinical deterioration.

Nutritional Consequences

Incorrect feeding tube placement also has important nutritional and healthcare consequences. Detection of malposition necessitates removal and reinsertion of the tube, delaying the initiation or continuation of enteral nutrition [29]. Repeated placement attempts may further increase this delay; in one clinical series, patients with malpositioned tubes required substantially more tube insertions than patients without complications (mean of 6.8 versus 2.2 insertions per patient) [26]. Such delays may contribute to inadequate nutrient and energy delivery, increasing the risk of malnutrition and impairing recovery, wound healing, immune function, and overall clinical outcomes.

However, the available evidence more directly supports an association between inadequate or delayed nutritional support and adverse clinical outcomes than a direct causal relationship between feeding tube repositioning itself and malnutrition or impaired wound healing. Nutritional support in malnourished or nutritionally at-risk medical inpatients has been associated with increased energy and protein intake and weight gain, and an updated meta-analysis of 27 trials involving 6,803 patients found associations with lower mortality and fewer nonelective hospital readmissions [30]. Evidence comparing early with delayed enteral nutrition also supports minimizing avoidable interruptions to nutritional therapy. A systematic review of 13 trials involving 1,173 patients undergoing gastrointestinal surgery found that early postoperative enteral feeding was associated with reduced mortality and examined outcomes including wound infection, intra-abdominal abscess, pneumonia, anastomotic leakage, and length of hospital stay [31]. These findings support the importance of timely nutritional provision, although they do not establish that a specific delay caused by feeding tube repositioning directly causes malnutrition, impaired wound healing, or prolonged hospitalization.

Repeated placement attempts and additional diagnostic procedures may further increase patient discomfort and expose patients to unnecessary radiation. Furthermore, complications related to tube malposition often prolong hospital stay, increase the need for additional medical and surgical interventions, and substantially elevate healthcare costs. Consequently, minimizing avoidable interruptions to nutritional therapy may help reduce nutritional deficits and associated adverse outcomes, particularly in patients who are already malnourished or at high nutritional risk [30]. Ensuring accurate radiological confirmation before feeding is therefore essential not only for patient safety but also for optimizing nutritional therapy and improving overall healthcare efficiency.

Advantages of radiological confirmation

Radiological confirmation remains the gold standard for verifying the position of blind-inserted enteral feeding tubes due to its high diagnostic accuracy. It enables reliable confirmation of correct tube placement and allows detection of complications, including malposition and inadvertent respiratory placement. International guidelines recommend radiographic confirmation before the initial use of enteral feeding tubes because it substantially reduces the risk of pulmonary feeding and associated adverse outcomes. This approach is applicable to all blind-inserted enteral tubes and remains the most widely accepted method for ensuring patient safety.

Limitations of radiological confirmation

Despite its diagnostic reliability, radiological confirmation has several limitations. These include exposure to ionizing radiation, delays in initiating enteral feeding while awaiting imaging, and increased healthcare costs. Accurate interpretation requires trained healthcare professionals with appropriate competency in reviewing tube position on radiographs. In addition, repeat imaging may be required when tube migration or displacement is suspected during ongoing use.

Current guideline recommendations

Professional organizations, including the American Society for Parenteral and Enteral Nutrition (ASPEN), European Society for Clinical Nutrition and Metabolism (ESPEN), and National Institute for Health and Care Excellence (NICE), continue to recommend radiographic confirmation before the first use of any blind-inserted enteral feeding tube [32-34].

Current recommendations include the following: (1) radiographic confirmation should be obtained before administration of enteral feeds, fluids, or medications through a newly inserted blind tube; (2) radiographs should be interpreted by appropriately trained healthcare professionals; (3) tube position should be clearly documented before tube use; (4) repeat imaging should be performed whenever tube displacement or migration is suspected.

These recommendations emphasize that accurate verification and clinician competency remain essential components of safe enteral nutrition practice.

Recent evidence (2020-2025): Artificial intelligence-assisted radiological verification of enteral feeding tube position

Artificial intelligence (AI), particularly deep learning and convolutional neural networks (CNNs), has emerged as a promising supportive technology for radiographic interpretation of enteral feeding tube position [35]. AI-based systems are designed to automatically identify feeding tubes on chest radiographs, detect potential malposition, and prioritize high-risk examinations for urgent clinical review [36]. These technologies aim to improve patient safety, reduce reporting delays, and support clinicians with varying levels of radiographic interpretation experience while maintaining appropriate radiologist oversight.

ASPEN Perspective

In 2021, ASPEN published a competency model addressing nasogastric and nasoenteric feeding tube placement and verification [37]. The document reaffirmed that a correctly obtained and accurately interpreted radiograph remains the gold standard for confirming the initial placement of blindly inserted feeding tubes [37].

ASPEN recognized the potential role of emerging technologies, including electromagnetic guidance systems, bedside ultrasound, camera-assisted placement methods, and AI-supported radiographic interpretation. However, these technologies were considered complementary approaches rather than replacements for established radiographic confirmation due to insufficient evidence for independent clinical use. The guideline also highlighted the importance of clinician education, training, and competency in radiograph interpretation to maintain safe practice.

ESPEN Perspective

ESPEN guidelines continue to recommend radiographic confirmation before the first use of blind-inserted enteral feeding tubes [38]. Recent reviews in clinical nutrition literature indicate that emerging technologies, including POCUS, electromagnetic tracking, real-time video-assisted placement, impedance-based systems, and AI-assisted imaging, may improve the safety and efficiency of tube placement [39].

However, current evidence remains insufficient to support AI as a standalone verification method. Conventional radiographic confirmation interpreted by trained professionals continues to represent the reference standard for clinical practice.

Evidence From Radiology Studies

Recent radiology research has evaluated AI algorithms for automated assessment of enteral feeding tube position on chest radiographs.

A multicenter study published by Drozdov et al. in 2023 developed a deep learning model capable of detecting nasogastric tube malposition on frontal chest radiographs [40]. The model demonstrated strong diagnostic performance, achieving an area under the receiver operating characteristic curve (AUC) of 0.90 (95% CI: 0.88-0.93) compared with expert radiologist consensus. Furthermore, when junior physicians used AI decision support, agreement with radiologists regarding decisions to commence feeding improved, with Cohen’s kappa increasing from 0.53 to 0.65. These findings suggest that AI may enhance clinical decision-making and reduce the risk of feeding through a misplaced tube.

A 2025 study published by Park et al. evaluated a dual-stage deep learning model designed to identify the pathway and final position of nasogastric tubes on chest radiographs [36]. The researchers reported that AI could improve automated recognition of tube malposition and assist with prioritization of abnormal examinations within radiology workflows, particularly in high-volume hospital environments.

Another study published in 2022 demonstrated that CNN models trained using weakly supervised learning approaches could improve assessment of enteral feeding tube position, even when only limited labelled imaging datasets were available [35]. These findings indicate that AI systems may achieve clinically useful performance despite challenges associated with obtaining large annotated datasets, supporting their potential future implementation across healthcare settings.

Workflow integration and clinical challenges

Although AI-assisted radiographic interpretation demonstrates promising diagnostic performance, integration into routine clinical practice presents several challenges [41]. Research published in 2024 examining human-centered AI implementation identified key considerations, including clinician trust, medicolegal responsibility, transparency of AI decision-making, handling of uncommon tube positions, and potential bias arising from training datasets [42].

The diagnostic performance of AI may also vary according to the amount and type of clinical information available. Recent work by Lotfian et al., including Suthar et al., demonstrated that the diagnostic accuracy of a large language model changed as additional case information was progressively provided, highlighting the importance of context and information completeness when evaluating AI-based diagnostic performance [43,44].

Additional challenges include variability in image quality and acquisition protocols, limited representation of uncommon clinical scenarios within training datasets, and uncertainty regarding the generalizability of AI performance when systems are applied to populations or clinical settings different from those used for development and validation [40,45].

Importantly, promising diagnostic performance in retrospective studies does not necessarily demonstrate improved patient outcomes, workflow efficiency, or patient safety in routine clinical practice. AI systems may produce both false-positive and false-negative assessments, while inappropriate reliance on automated outputs may introduce additional safety risks. Furthermore, concerns regarding reliability, accountability, trust, governance, and the potential for systemic errors remain important barriers to implementation [45].

Current evidence supports the role of AI as a clinical decision-support tool rather than an autonomous replacement for radiologists. Effective implementation will require careful validation, appropriate workflow integration, and continued professional oversight.

Future directions

Future developments are likely to integrate AI systems with digital radiography platforms, picture archiving and communication systems (PACS), and electronic health records to enable real-time alerts for incorrectly positioned feeding tubes. AI may assist with rapid triage of urgent radiographs, reduce reporting delays, and improve consistency in tube position assessment [46]. Nevertheless, these potential benefits should be distinguished from demonstrated clinical benefit. Further research is needed to establish whether AI implementation improves patient safety, reduces clinically significant errors, or produces measurable improvements in workflow efficiency. Performance should also be assessed in technically limited radiographs, uncommon tube positions, and patient populations that may be under-represented in development datasets.

However, large prospective multicenter validation studies remain necessary before AI-assisted verification can be incorporated into international guidelines as a replacement for conventional radiographic interpretation.

## Conclusions

Radiological confirmation remains the gold standard for verifying enteral feeding tube placement before initiating enteral nutrition. It provides reliable detection of correct tube position and prevents serious complications associated with malposition, including aspiration pneumonia, pulmonary injury, and death. Although emerging technologies such as AI, ultrasound, and electromagnetic guidance show promise, they currently serve as supportive tools rather than replacements for radiographic confirmation. Accurate imaging interpretation, clinician competency, and adherence to evidence-based guidelines remain essential to ensure safe and effective enteral nutrition practice.
